# Coronavirus Disease 2019 Related Clinical Studies: A Cross-Sectional Analysis

**DOI:** 10.3389/fphar.2020.540187

**Published:** 2020-09-02

**Authors:** Lin-Lu Ma, Xuan Yin, Bing-Hui Li, Jia-Yu Yang, Ying-Hui Jin, Di Huang, Tong Deng, Yun-Yun Wang, Xue-Qun Ren, Jianguang Ji, Xian-Tao Zeng

**Affiliations:** ^1^Center for Evidence-Based and Translational Medicine, Zhongnan Hospital of Wuhan University, Wuhan, China; ^2^Department of Rehabilitation, Taihe Hospital, Hubei University of Medicine, Shiyan, China; ^3^Institutes of Evidence-Based Medicine and Knowledge Translation, Henan University, Kaifeng, China; ^4^Administrative Office of Hospital Director, Zhongnan Hospital of Wuhan University, Wuhan, China; ^5^Center for Primary Health Care Research, Lund University/Region Skåne, Malmö, Sweden

**Keywords:** coronavirus disease 2019, SARS-CoV-2, clinical trial, registration, *ClinicalTrials.gov*

## Abstract

**Objective:**

The quality and rationality of many recently registered clinical studies related to coronavirus disease 2019 (COVID-19) needs to be assessed. Hence, this study aims to evaluate the current status of COVID-19 related registered clinical trial.

**Methods:**

We did an electronic search of COVID-19 related clinical studies registered between December 1, 2019 and February 21, 2020 (updated to May 28, 2020) from the *ClinicalTrials.gov*, and collected registration information, study details, recruitment status, characteristics of the subjects, and relevant information about the trial implementation process.

**Results:**

A total of 1,706 studies were included 10.0% of which (n=171) were from France, 943 (55.3%) used an interventional design, and 600 (35.2%) used an observational design. Most of studies (73.6%) aimed to recruit fewer than 500 people. Interferon was the main prevention program, and antiviral drugs were the main treatment program. Hydroxychloroquine and chloroquine (230/943, 24.4%) were widely studied. Some registered clinical trials are incomplete in content, and 37.4% of the 1,706 studies may have had insufficient sample size.

**Conclusion:**

The quality of COVID-19 related studies needs to be improved by strengthening the registration process and improving the quality of clinical study protocols so that these clinical studies can provide high-quality clinical evidence related to COVID-19.

## Introduction

COVID-19, which broke out at the beginning of 2020, has spread rapidly (Zhou P. et al., 2020). Its clinical manifestations are very similar to Severe Acute Respiratory Syndrome (SARS). In severe cases, patients may go on to develop acute respiratory distress syndrome (ARDS). Patients with severe COVID-19 need intensive care to decrease mortality (Huang et al., 2020). As of July 13, 2020, there have been more than 12.8 million confirmed cases and 568,000 deaths globally (Johns Hopkins University, 2020).

COVID-19 is an emerging infectious disease for which, there is no specific treatment to date. Healthcare professionals have only been able to alleviate patients’ symptoms based on their experience (Jin et al., 2020) as up to now they have had insufficient knowledge of this disease. Hence, randomized clinical trials (RCTs) are necessary to verify the safety and effectiveness of the proposed drugs. Many scientists and clinicians have conducted clinical investigations, diagnostic accuracy tests, and treatment evaluations to understand the progress of COVID-19 and to improve clinical diagnosis and treatment. It is thus essential to evaluate the rationality and the potential value of proposed clinical trials because so many studies have emerged in such a short period and some of them might lack scientific value. Therefore, we performed this survey in order to have a comprehensive understanding of the current clinical trials related to COVID-19.

## Methods

This study analyzed the characteristics of the clinical studies of COVID-19 registered in *ClinicalTrials.gov* (https://clinicaltrials.gov/) between December 1, 2019 and February 21, 2020 (updated to May 28, 2020). All COVID-19 related studies, including etiology, risk factors, prevention, diagnosis, treatment, prognosis, and psychology were included. The search terms were: 2019-nCoV, 2019 novel coronavirus, novel coronavirus pneumonia, COVID-19, coronavirus disease 2019, SARS-CoV-2.

We extracted the following information from registered studies: registration number, registration date, registration title, primary sponsor, funding source, study type, study phase, study objectives, study design, length of the study, intervention, countries of recruitment and research settings, recruiting status, allocation, sample size, participant age, gender, masking, the time and method of sharing individual participant data (IPD), data management committee.

Descriptive statistics were used to summarize the characteristics of all included clinical studies. Categorical variables were expressed as percentages and frequencies. All data were summarized using Microsoft Excel 2019.

## Results

### General Characteristics of the Included Studies

**Figure 1:**
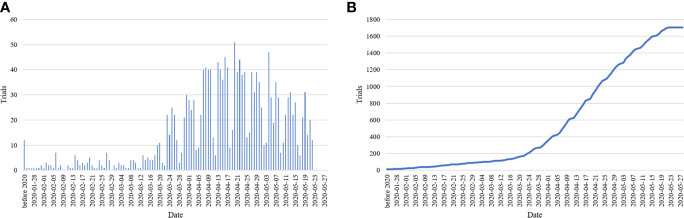
Bar chart of registered studies per day **(A)** and cumulative sum chart of registered studies **(B)**.

A total of 1,706 studies were included. Among these clinical studies (**Table 1**), the first one was registered on January 23, 2020, and the number of trials registered daily subsequently increased, peaking at 51 in a single day (**Figure 1**). For the total study period, 73.8% studies (n = 1259) planned to continue for less than 12 months and 25.1% more than 12 months. Of them, 943 (55.3%) used an interventional design and 600 (35.2%) used an observational design. As for the recruitment status, 82 (4.8%) studies had completed recruitment, 922 (54.0%) were recruiting, and 683 (40.0%) had not yet started recruiting, while some others were terminated/withdrawn (n = 12, 0.7%) or suspended (n = 7, 0.4%). For sample sizes, most of them (n = 1255, 73.6%) aimed to recruit less than 500 participants, 6.5% (n = 111) recruited 100 to 499 participants, 18.9% recruited more than 1,000, and 1.1% (n = 18) studies did not specify the number of participants recruited. Almost all studies recruited both males and females (n = 1662, 97.4%), 83.1% studies (n = 1417) included adults and only 16.9% (n = 289) involved children.

**Table 1:** General characteristics of the included studies.

	Number	Percentage
Total	1706	100%
*Length of study time*		
0 <L≤6m	816	47.8%
6m<L≤12m	443	26.0%
12m<L≤18m	154	9.0%
L>18m	274	16.1%
NA	19	1.1%
*Study type*		
Interventional	943	55.3%
Observational	600	35.2%
Diagnostic Test	145	8.5%
Expanded Access	18	1.1%
*Recruitment status*		
Recruiting	922	54.0%
Not yet recruiting	683	40.0%
Completed	82	4.8%
Terminated/Withdrawn	12	0.7%
Suspended	7	0.4%
*Enrollment*		
<100	638	37.4%
100–499	617	36.2%
500–999	111	6.5%
≥1000	322	18.9%
NA	18	1.1%
*Sex/Gender*		
Both	1662	97.4%
Only female	35	2.1%
Only male	7	0.4%
NA	2	0.1%
*Ages*		
Child	20	1.2%
Adult	1417	83.1%
Child and Adult	269	15.8%

NA, Not available. Child means a person under the age of 18.

### Methodological Quality of the Included Studies

Among the 943 interventional studies, the primary purpose was treatment of the disease (n = 714, 75.7%). Seven hundred eighty-one (82.8%) were designed with at least two groups, most commonly parallel assignment (n = 717, 76.0%). Seven hundred twenty (76.4%) were randomized and 78 (8.3%) were non-randomized. More than 56.2% studies (n = 530) were open label, and only 33.0% being double, triple, or quad-masked. As for the 600 observational studies, 376 (62.7%) were cohort studies, and 377 (62.8%) were prospective design. For the 145 diagnostic studies, 32 studies (22.1%) focused on imaging studies, 36 studies (24.8%) focused on nucleic acid detection, and 15 studies (10.3%) focused on specific antibody. Details are shown in **Table 2**.

**Table 2:** Trials design data.

Study Type		Number	Percentage	
Interventional	Total	943	100%	
	*Number of Arms*			
	1	161	17.1%	
	2	622	66.0%	
	3	83	8.8%	
	4	47	5.0%	
	> 4	29	3.1%	
	NA	1	0.1%	
	*Allocation*			
	Randomized	720	76.4%	
	Non-Randomized	78	8.3%	
	NA	145	15.4%	
	*Intervention Model*			
	Single Group Assignment	160	17.0%	
	Parallel Assignment	717	76.0%	
	Sequential Assignment	33	3.5%	
	Factorial Assignment	12	1.3%	
	Crossover Assignment	21	2.2%	
	*Masking (Blinding)*			
	Open Label	530	56.2%	
	Single	102	10.8%	
	Double	109	11.6%	
	Triple	66	7.0%	
	Quadruple	136	14.4%	
	*Primary Purpose*			
	Treatment	714	75.7%	
	Prevention	124	13.1%	
	Others	105	11.1%	
	Phases			
	Early Phase 1	18	1.9%	
	Phase 1	47	5.0%	
	Phase 1|Phase 2	54	5.7%	
	Phase 2	265	28.1%	
	Phase 2|Phase 3	76	8.1%	
	Phase 3	174	18.5%	
	Phase 4	56	5.9%	
	NA	253	26.8%	
Observational	Total	600	100%	
	*Observational Model*			
	Case-Only	52	8.7%	
	Case-Control	74	12.3%	
	Cohort	376	62.7%	
	Other	98	16.3%	
	*Time Perspective*			
	Retrospective	100	16.7%	
	Prospective	377	62.8%	
	Cross-Sectional	67	11.2%	
	Other	56	9.3%	
Diagnostic Test	Total	145	100%	
	Imaging exams	32	22.1%	
	nucleic acid detection	36	24.8%	
	IgM/IgG	15	10.3%	
	Other	62	42.8%	

NA, Not available.

### Detailed Characteristics of Included Studies

Of the 1,706 studies, 1,200 (70.3%) were initiated by researchers from hospitals, universities, or scientific research institutions; whereas a few (9.8%) were initiated by companies, and 338 (19.8%) were funded by others, such as individuals or community-based organizations. The highest number of studies were conducted in France (n = 171, 10.0%) and the second highest in the United States (n = 108, 6.3%). Of the 1,706 studies, only 33 studies (1.9%) were funded by National Institutes of Health (NIH) or U.S. Federal agencies, 255 (14.9%) were funded by pharmaceutical or device companies, and 83.1% were funded by others, such as individuals, universities, or community-based organizations. Six hundred ten (35.8%) clearly reported the existence of a data monitoring committee, and 192 (11.3%) had IPD sharing statement. Details are shown in **Table 3**.

**Table 3:** Sponsor, location, and data monitoring characteristics of the included studies.

	Number	Percentage
Total	1706	100%
*Study Sponsor*		
Hospital	593	34.8%
University	476	27.9%
Industry	168	9.8%
Research Institution	131	7.7%
Other	338	19.8%
*Collaborators*		
Has Collaborators	621	36.4%
No Collaborators	1085	63.6%
*Places to recruit and conduct research*		
France	171	10.0%
United States	108	6.3%
Italy	52	3.0%
China	43	2.5%
United Kingdom	35	2.1%
Spain	33	1.9%
Germany	29	1.7%
Egypt	25	1.5%
Other	154	9.0%
NA	1094	64.1%
*Funder Type*		
NIH	27	1.6%
U.S. Fed	6	0.4%
Industry	255	14.9%
Other	1418	83.1%
*Data Monitoring Committee*		
Has Data Monitoring Committee	610	35.8%
Not have Data Monitoring Committee	811	47.5%
NA	285	16.7%
*IPD Sharing Statement*		
Yes	192	11.3%
No	731	42.8%
Undecided	334	19.6%
NA	449	26.3%

NA, Not available.

### Description of Drugs in the Included Interventional Studies

Among the 943 interventional studies, 416 studies (44.1%) explored the effectiveness and/or safety of drugs commonly used in preventing and treating COVID-19, such as hydroxychloroquine (HCQ), chloroquine (CQ), immunotherapy (including stem cell therapy, monoclonal antibody, immunoregulation), lopinavir/ritonavir, glucocorticoids, interferon, targeted therapy (Baricitinib, Ruxolitinib, Imatinib), favipiravir, and Remdesivir. In addition, 66 studies (7.0%) focused on convalescent plasma. Other interventions, such as dietary supplements, devices and behavioral programs, accounted for 48.9%. Details are shown in **Table 4**.

**Table 4:** Interventional clinical studies for COVID-19.

Interventions	Trials Number	Percentage
Total	943	100%
Hydroxychloroquine	157	16.6%
Hydroxychloroquine & Azithromycin	51	5.4%
Chloroquine	22	2.3%
Remdesivir	10	1.1%
Lopinavir/Ritonavir	32	3.4%
Favipiravir	16	1.7%
Interferon	23	2.4%
Glucocorticoid	29	3.1%
Immunity therapy	53	5.6%
Targeted Therapy	23	2.4%
Convalescent plasma	66	7.0%
Dietary Supplement	18	1.9%
Device	63	6.7%
Behavioral	47	5.0%
Other	333	35.3%

## Discussion

The COVID-19 epidemic is still raging around the world. Exploring the characteristics of registered clinical studies related to COVID-19 and clarifying the direction of further research can help reduce the potential disease burden of COVID-19 (Gupta et al., 2020). There was a cross-sectional study that reviewed the drug and plasma registration trials in March 2020, characterizing the scope, objectives and content of clinical studies (Mehta et al., 2020). With the rapid increase in registration research, the status of registration studies may also change. This survey conducted a comprehensive summary of COVID-19 related studies registered in the *ClinicalTrials.gov* as of May 28, 2020. Results showed that most studies with an interventional design were aimed at adult participants, and were conducted using multicenter, randomized, parallel assignments, and open-label methods. A systematic review showed that compared with adults, children with COVID-19 have a milder disease course, with better prognosis and extremely low mortality (Ludvigsson, 2020). As a result, only 16.9% of registered studies involved children. As a factor of disease outcomes (Hou et al., 2019), only 2.5% studies focus on the participants’ gender. The included clinical studies involved disease prevention, diagnostic accuracy, drug treatment, medical devices, prognosis, as well as treatment of critical COVID-19. A number of these studies (n=638, 37.4%) may have had insufficient sample size.

Registration of COVID-19 related clinical studies is ongoing. The underlying methodological quality limitations of these clinical studies should be noted, such as lack of control group, insufficient sample size, or non-randomization, which might preclude drawing concrete conclusions (Bauchner and Fontanarosa, 2020; Ma et al., 2020). Our results found that nearly half of the registered trials did not exceed 6 months, and 37.4% of the registered trials recruited less than 100 people. The inclusion of less than 100 people does not automatically indicate that the study results are unreliable. Different studies need to estimate sample size according to outcomes. More studies are needed which use samples based on the estimated sample size. Insufficient or under-estimated sample size is a major shortcoming of the current clinical trials, which can cause false negative or false positive results, reduce credibility, and even have catastrophic consequences (Ruberg and Akacha, 2017). Therefore, although some studies had reported that some interventions may shorten intubation time, hospitalization time or reduce mortality; these findings did not represent the actual therapeutic effect of the drug (Gautret et al., 2020). The outbreak of the epidemic may pressurize researchers to quickly find targeted therapeutic drugs which are effective in the short term. However, if the length of the study was too short, it might preclude carrying out multiple follow-ups on the patients, and the long-term effect index of drug treatment cannot be obtained.

Our results found that of the intervention study 82.8% of the registered trials were designed for at least two groups, 76.4% were assigned randomly, 56.2% were open label, and 75.7% were mainly for treatment. Of the observational studies, most utilized cohorts (62.7%) and prospective (62.8%) designs. Randomization can largely avoid confusion and reduce selection bias in treatment comparison (Sessler and Imrey, 2015). However, RCTs often require large sample size, long research duration, incur high costs and may also be difficult to implement. At this time, adaptive trial design can usually be adopted (Bhatt and Mehta, 2016). However, it should be noted that observational research will be accompanied by some biases and limitations, and it is necessary to interpret the test results carefully (Shang et al., 2020). Besides, some of these studies did not have a control group or lack a real “control”, which will limit the effective inferences that can be drawn. There is a need for rigorous design and attention to trial protocols for research drug management to discover the true efficacy of interventions (Bauchner and Fontanarosa, 2020).

More and more researchers realize that clinical trials need to be registered before the recruitment, and registration is beneficial for sharing clinical trial information and reducing publication bias (Aslam et al., 2013). It is understandable that clinical trials must be launched and implemented quickly due to the sudden COVID-19 epidemic; however, a properly designed clinical trial is still the core to provide scientific evidence and achieve clinical conclusions. Randomized controlled trials are considered to be the highest quality clinical research methods, and random sequence generation, blinding, and allocation concealment during the implementation of the study are critical to the success of the study (Schulz and Grimes, 2002b; Schulz and Grimes, 2002a; Sessler and Imrey, 2015). It is thus essential for clinical trials to be designed by a professional team to meet the requirements of a successful study before registration. An appropriate research design should be selected according to the research purpose, with sample size being estimated in advance, and timely submission of the research plan to the ethical committee to avoid deficiencies. At the time of registration, the person responsible for the registration should have a comprehensive understanding of the characteristics of the study protocol and clinical trial, so as not to cause confusion to other researchers due to the ambiguity of registration content, such as countries of location, presence or absence of data monitoring committee. We found some registered clinical trials have incomplete content. Therefore, clinical trial registration agencies should strengthen supervision of trial registration. After the study completion, collation and strict statistical analysis the researchers should upload the resulting data to the registration agency in a timely manner (Goldacre, 2017). IPD sharing helps to accelerate the conversion of clinical resources and promote scientific breakthroughs. Hence, we call on researchers to share IPD to promote transparency, so that effective conclusions drawn from trials can be quickly applied to control the epidemic, and to provide a basis for COVID-19 prevention and treatment.

COVID-19 is a new infectious disease, which has affected health insurance (Gheorghe et al., 2019), and its underlying mechanisms of transmission and pathogenesis are still being explored. High quality clinical studies are the basis of clinical practice guidelines, especially WHO’s emergency guidelines (Norris et al., 2019). Some clinical trials focus on the prevention of COVID-19. It is widely believed that SARS-CoV-2 is transmitted through respiratory droplets and by close contact (Jin et al., 2020). Earlier studies have shown that masks are very effective for filtering influenza viruses (Zhou et al., 2018). However, there are no clinical study results that can prove that wearing masks can prevent COVID-19. A study has analyzed the pandemic trends and mitigation measures of COVID-19 in Wuhan, China, Italy, and New York City. Results showed that the difference between with and without facial masks represents the determinant of pandemic trends in the three epicenters. The authors thought that wearing a mask is the most effective way to prevent interpersonal transmission in public places (Zhang R. et al., 2020). In hospitals, healthcare professionals are at greater risk of exposure to SARS-CoV-2 than public. A multi-center RCT (Registration number: *NCT04296643*) from Canada is expected to recruit 576 nurses to compare and analyze the preventive effects of medical masks with N95 respirators on COVID-19. In addition, some clinical studies have focused on the preventive effects of drugs for COVID-19, such as CQ and HCQ. Chloroquine and hydroxychloroquine are both antimalarial drugs, and the mechanism of preventing and treating COVID-19 is not yet clear. Some researchers thought CQ and HCQ may confer antiviral effect at the pre-infection stages (Zhou D. et al., 2020). However, the possible cardiac side effects caused by the combination of CQ or HCQ and AZ, such as prolonged QT interval must be considered. Hence, clinical studies are needed to confirm the preventive effect of CQ or HCQ on COVID-19 (Registration number: *NCT04303507, NCT04334148*).

An accurate diagnosis is the fundamental prerequisite for efficient control of COVID-19. We included 145 clinical studies exploring the diagnosis of COVID-19. These diagnostic accuracy tests mainly focus on imaging examination, nucleic acid detection, and IgM/IgG. Detection of SARS-CoV-2 RNA by reverse-transcription polymerase chain reaction (RT-PCR) is the most commonly used to diagnose COVID-19. Early studies have shown that RT-PCR has relatively poor sensitivity, and false negative test results will miss some potential infected persons, which has a huge impact (Fang et al., 2020). Furthermore, the standard RT-PCR test takes about 3 h to complete. The cost of each test is about $10. The high cost per test may limit the number of tests (Esbin et al., 2020). Hence, researchers wanted to design some test kits in order to detect SARS-CoV-2 quickly and conveniently (Chu et al., 2020; Shirato et al., 2020; To et al., 2020; Yu et al., 2020). COVID-19 patients also have some typical computed tomography (CT) manifestations, such as ground glass opacities (Fang et al., 2020; Lu et al., 2020; Zhang J. J. et al., 2020). As a fast and effective method, CT can be used for auxiliary diagnosis. However, it should be noted that some patients may have atypical CT imaging manifestations (Jin et al., 2020; Lu et al., 2020; Wang W. G. et al., 2020). In addition, as the product of human immune system reaction to SARS-CoV-2, IgM/IgG can provide information about the course of the virus infection over time and provide the basis for the diagnosis of COVID-19. Some researchers have developed an IgM-IgG combined antibody test kit with a sensitivity of 88.66% and a specificity of 90.63%, but there were still false negative and false positive results (Li et al., 2020). The sensitivity and specificity of the IgM/IgG rapid diagnostic kit are currently being evaluated in some studies (*Registration number: NCT04346186, NCT04348864*).

Drug treatment is a very important part of the registration studies. Few drugs were used to treat COVID-19, such as CQ, HCQ, IFN, lopinavir/ritonavir, Oseltamivir, Umifenovir, dexamethasone. There is currently no clear evidence that these drugs are specific drugs for the treatment of COVID-19 other than dexamethasone (Gautret et al., 2020; RECOVERY Collaborative Group, 2020; Tang et al., 2020). The RECOVERY trial claims that dexamethasone can reduce the risk of death for patients on ventilators (RR 0.64; 95% CI, 0.51 to 0.81) and patients on oxygen (RR 0.82; 95% CI, 0.72 to 0.94) (RECOVERY Collaborative Group, 2020). The National Institutes of Health recommends the use of dexamethasone to treat COVID-19 patients who require supplemental oxygen in its guidelines (COVID-19 Treatment Guidelines Panel, 2020). As a new experimental broad-spectrum antiviral medication, Remdesivir is considered to be effective in inhibiting the replication of SARS coronavirus and MERS coronavirus. Two RCT studies showed that compared with placebo, the use of Remdesivir could shorten the recovery time of patients with COVID-19 (Beigel et al., 2020; Wang Y. et al., 2020). As of June 2020, it has been authorized for emergency treatment of COVID-19 in the US, Singapore, Japan, and the UK. CQ was first used to treat malaria, HCQ as its analogue is less toxic than CQ. CQ/HCQ is other drugs under consideration for treating COVID-19. So far, the drugs have been controversial. Some studies have shown that the drugs have significant efficacy in alleviating symptoms (Sarma et al., 2020; Tang et al., 2020), but some studies have reported that CQ/HCQ has potential cardiac side effects, such as prolonging QT interval (Borba et al., 2020). In June 2020, the U.S. Food and Drug Administration revoked the emergency use authorization for HCQ. A clinical trial to evaluate the safety and effectiveness of HCQ for the treatment of COVID-19 has been stopped by the NIH. After its fourth interim analysis, the data and safety monitoring board concluded that while there was no harm, HCQ was unlikely to be beneficial to hospitalized COVID-19 patients (NIH, 2020). In July 2020, WHO discontinued the Solidarity Trial’s HCQ and lopinavir/ritonavir arms. Although lopinavir/ritonavir can reduce SARS-CoV-2 viral loads (Lim et al., 2020), the Solidarity Trial’s interim results showed that compared with standard treatment, HCQ and lopinavir/ritonavir produce little or no reduction in the mortality of hospitalized COVID-19 patients (WHO, 2020). IFN has been used to treat SARS and MERS, and can improve patient survival (Haagmans et al., 2004; Mustafa et al., 2018); Liu et al. (Liu et al., 2020) reported that the efficacy is not clear for the treatment of COVID-19 using IFN. Hence, a clinical trial has been investigating the efficacy of IFN for the treatment COVID-19 (*Registration number: NCT04254874*). A study (Tian et al., 2020) reported a new coronavirus-specific human monoclonal antibody—CR3022, which can bind SARS-CoV-2 receptor-binding domain, and has potential function to prevent and treat SARS-CoV-2 infections. In addition, there have been some clinical studies investigating the convalescent plasma for the treatment of COVID-19.

A few limitations should be noted in this study. Because COVID-19 is a new disease, its name as well as the name of virus changed many times, so there may be a small number of studies using other names for the registration, which may not have been retrieved. Additionally, due to the worldwide spread of COVID-19, studies will continue to be registered every day and the number of clinical studies is growing, which may also cause some bias. In addition, this study only retrieved trials registered in *ClinicalTrials.gov*. Although *ClinicalTrials.gov* includes more than 3.4 million research studies in 214 countries, some studies may not have been registered on this platform.

In conclusion, the number of registered COVID-19 related clinical studies has increased rapidly since the outbreak, involving epidemiology, risk factors, prevention, diagnosis, treatment, rehabilitation, and psychological aspects. However, some registration parameters are not complete, so it is necessary to strengthen the registration monitoring and supervision for providing high-quality clinical evidence.

## Data Availability Statement

The datasets generated for this study are available on request to the corresponding authors.

## Author Contributions

X-TZ and JJ take responsibility for the integrity of the data and the accuracy of the data analysis. Concept and design: X-TZ, X-QR, and JJ. Acquisition, analysis, or interpretation of data: All authors. Drafting of the manuscript: L-LM, B-HL, and DH. Critical revision of the manuscript for important intellectual content: All authors. Statistical analysis: XY, Y-YW, and J-YY. Administrative, technical, or material support: Y-YW, B-HL, and XY. Supervision and review: X-TZ, X-QR, and JJ.

## Funding

This work was supported (in part) by the National Key Research and Development Program of China (2020YFC0845500).

## Conflict of Interest

The authors declare that the research was conducted in the absence of any commercial or financial relationships that could be construed as a potential conflict of interest.
